# Acute supratentorial subdural hematoma after craniocervical junction arachnolysis in a patient with posttraumatic syringomyelia; case report and literature review

**DOI:** 10.1002/ccr3.7170

**Published:** 2023-03-31

**Authors:** Keyvan Eghbal, Majid Reza Farrokhi, Seyed Reza Mousavi, Mohammadhadi Amir Shahpari Motlagh, Ali Kazeminezhad, Fariborz Ghaffarpasand

**Affiliations:** ^1^ Department of Neurosurgery Shiraz University of Medical Sciences Shiraz Iran; ^2^ Shiraz Neurosciences Research Center, Department of Neurosurgery Shiraz University of Medical Sciences Shiraz Iran; ^3^ Department of Neurosurgery, Peymanieh Hospital, Trauma Research Center Jahrom University of Medical Sciences Jahrom Iran; ^4^ Research Center for Neuromodulation and Pain Shiraz University of Medical Sciences Shiraz Iran

**Keywords:** acute subdural hematoma, arachnolysis, craniovertebral junction, spinal adhesive arachnoiditis

## Abstract

In patients with SAA rapid CSF drainage while performing durotomy must be avoided by utilizing cotton pads and lowering the head level to avoid catastrophic complications.

## 1 INTRODUCTION

A 37‐year‐old woman with chronic progressive myelopathy and syringomyelia and scoliosis presented with difficulty in walking at presentation. She suffered from spinal adhesive arachnoiditis in craniovertebral junction leading that underwent posterior fossa decompression and developed subsequent supratentorial subdural hematoma formation requiring surgical evacuation.

Spinal adhesive arachnoiditis (SAA) is characterized by collagen deposition in pia matter of arachnoid space due to inflammation caused by various etiologies.^1^ Trauma, infection, subarachnoid hemorrhage (SAH), and iatrogenic (surgery or epidural/subdural injections) are among the most common causes of SAA.^1^, ^2^, ^3^ SAA is also referred as foramen magnum arachnoiditis (FMA), basal arachnoiditis, hindbrain arachnoiditis, chronic arachnoiditis at the foramen magnum, and arachnoiditis at the craniocervical junction (CVJ).^3^, ^4^, ^5^ Clinically, the condition is associated with progressive myelopathy, quadriparesis, spastic gate, syrinx formation, myelopathy, and radicular symptoms.^1^, ^2^, ^5^, ^6^, ^7^, ^8^ It has been reported that syrinx formation is secondary to cerebrospinal fluid (CSF) accumulation distal to its normal passage through central canal or spinal cord interstitial space.^9^, ^10^, ^11^, ^12^

Intracranial hematoma, remote from surgical site, has been reported in literature.^13^ These include epidural (EDH), subdural (SDH), or intraparanchymal hematomas following various brain or spine surgeries.^13^ Acute supratentorial SDH following posterior fossa surgery or CSF diversion surgeries has rarely been reported before.^6^, ^7^, ^14^, ^15^, ^16^, ^17^, ^18^, ^19^, ^20^, ^21^, ^22^ We herein present a case of acute supratentorial SDH following arachnolysis of delayed posttraumatic CVJ adhesive arachnoiditis.

## 2 CASE PRESENTATION

### 2.1 History and physical examination

A 37‐year‐old single woman from southern Iran was referred to our clinic due to progressive spastic gate and apraxia along with progressive thoracolumbar scoliosis for the past 6 months prior to presentation. She had no significant past medical, family or drug history except a falling object head trauma 5 years earlier. In physical examination, she had scoliotic thoracic spine and lower extremity proximal weakness (3/5). She also had right upper extremity radicular pain and proximal weakness (4/5). Plantar reflexes were upward bilaterally along, the Hoffmann's sign was positive and the deep tendon reflexes (DTR) were exaggerated (3/4). She could walk only with assistance utilizing a walker. Fundoscopic examination revealed normal optic disc borders without papilledema.

### 2.2 Imaging

**FIGURE 1:**
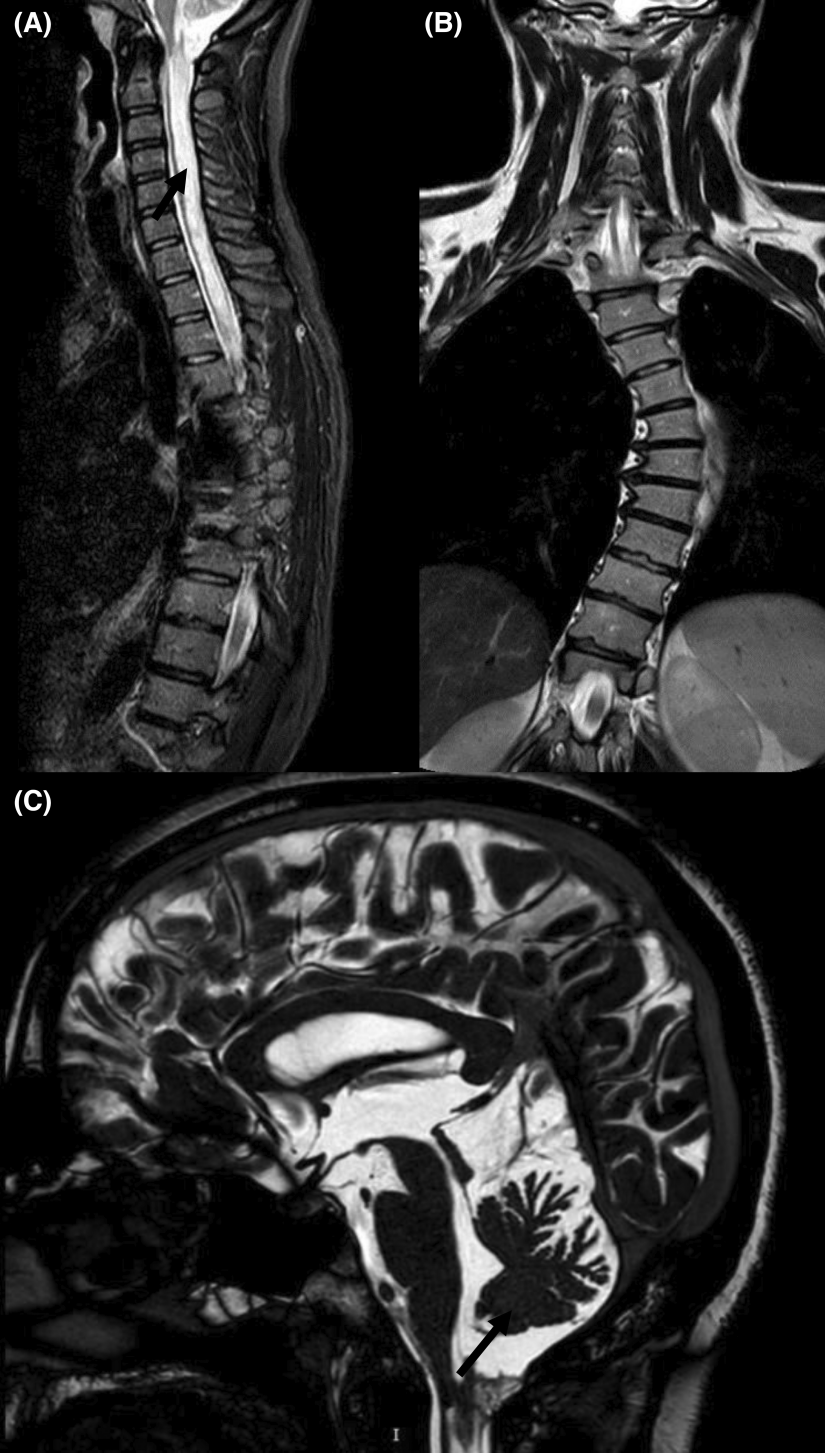
Sagittal T2‐weighted MRI of the cervicothoracic spine demonstrating a large holocord syrinx (arrow) extending from C2 to the conus medullaris (A); coronal T2‐weighted MRI of thecervicothoracic spine demonstrating a large S‐shape scoliosis with lower curve at L1 with convexity to right and upper curve at T6 with convexity to left (B); sagittal T2‐wighted MR images of the brain demonstrating severe adhesive arachnoiditis (arrow) and CSF blockade in craniocertebral junction (CVJ) (C).

Whole spine magnetic resonance imaging (MRI) was conducted revealing a large syrinx extending from C2 to lower thoracic levels (Figure 1A), a large S‐shape scoliosis with lower curve at L1 with convexity to right and upper curve at T6 with convexity to left (Figure 1B). Brain MRI, revealed a septate cystic mass in dorsal aspect of CVJ, pushing fourth ventricle and uppermost cervical spinal cord segments, causing signal changes (Figure 1C). A CVJ junction Cine MRI, revealed a septate thin‐wall cystic structure (18*12*9 mm) in posterior aspect of CVJ, causing CSF flow compromise, in association with extensive syringomyelia, without any evidence of hydrocephalus. There was normal CSF flow in anterior aspect of CVJ. Delayed post‐traumatic CVJ adhesive arachnoiditis, compressing fourth ventricle, brainstem and upper cervical spinal cord, causing alterations in CSF circulation and dynamics, was diagnosed and the patient was scheduled for CVJ decompression, durotomy, and arachnolysis in an elective setting.

### 2.3 Surgery

In standard prone position with her head fixed in Mayfield head‐holder and slight neck flexion, the patients underwent bilateral suboccipital craniotomy. About 3 × 3 cm midline suboccipital craniectomy, including dorsal rim of Foramen Magnum, in addition to C1 partial laminectomy was done. Dura was opened in linear fashion, 5‐mm from upper edge of craniectomy down to C1 lamina. There was a collection at dorsal aspect of CVJ, covering with numerous thick arachnoid webs, distorting normal anatomy of neurovascular structures in the area, without normal pulsation of arachnoid membranes. Then, microscopic arachnolysis with preservation of tethered arteries and nerves in arachnoid membranes was performed until floor of fourth ventricle was seen. The cisterna magna was also opened and the CSF normal flow was restored. The arachnolysis was continued caudally to reach normal dorsal spinal SAS. After microscopic coagulation of arachnoid webs with preservation of neurovascular structures, direct passage of CSF from fourth ventricle to spinal SAS was confirmed. The Duroplasty with autologous fascial graft was performed and the wound was closed.

### 2.4 Postoperative course

**FIGURE 2:**
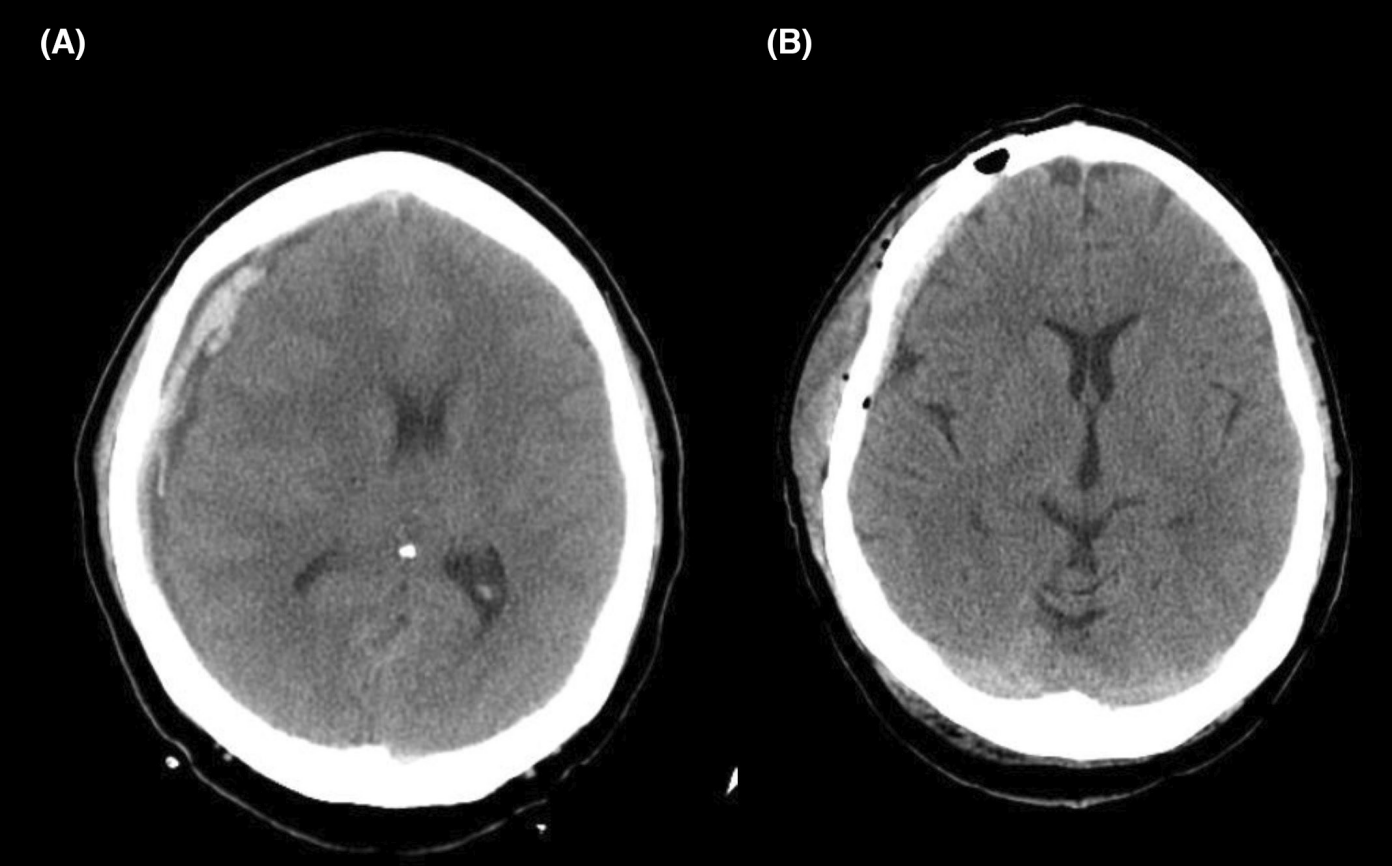
Axial brain CT‐scan of the patient after CVJ decompression revealing right frontoparietal acute sundural hematoma with 5 mm midline shift (A); postoperative axial brain CT‐scan of the patient demonstrating complete evacuation of the right subdural hematoma without midline shift (B).

**FIGURE 3:**
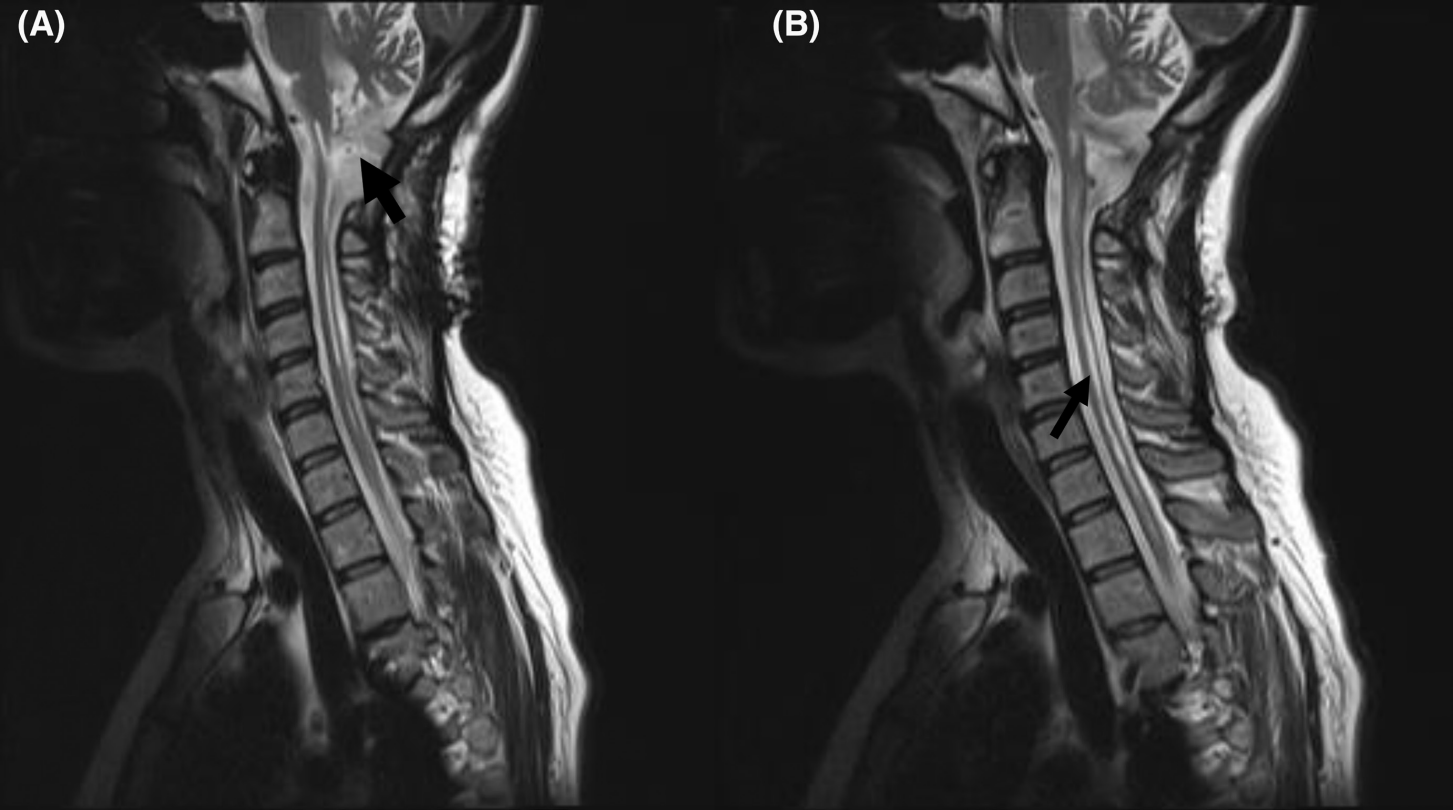
Sagittal T2‐weighted MRI of the cervicothoracic spine demonstrating suboccipital craniotomy and removal of the C1 arc with free CSF flow in the CVJ (arrow) (A); sagittal T2‐wighted MR images of the cervicothoracic juction demonstrating decreased size and diameter of the holocord syrinx when compared to preoperative images (arrow) along with normal CSF flow (B).

We had no major bleeding or hemodynamic catastrophic events during surgery. A postoperative brain computed tomography (CT) scan was performed before transferring to intensive care unit (ICU) revealing right frontoparietal (FP) acute SDH with nearly 5‐mm right to left midline shift (Figure 2A). The patient was transferred to operation room for surgical evacuation of SDH. The GCS score was 7/15 and anisocoria (right: 6 mm and left: 3 mm) was detected. The patient underwent right frontoparietal craniotomy for SDH evacuation. About 40 cc acute SDH was evacuated and cranioplasty was performed. The postoperative brain CT‐scan was acceptable (Figure 2B). The postoperative MR images of the CVJ revealed that the CSF flow was restored and the CSF blockade was extensively alleviated being associated with decreased size of the cervical syrinx (Figure 3A,B).

### 2.5 Outcome

The patient retained his normal consciousness a day after surgery and pupillary reflexes were bilateral and equal. She developed CSF leakage from CVJ surgical site at the fourth postoperative day, that was managed with insertion of lumbar drainage tube and medications. After 3 weeks of postoperative care, neurological examination improved significantly and she could walk without assistance. Her right upper extremity radicular pain and weakness were also resolved. She was discharged with both surgical wounds healed, having no sign of infection, no CSF leakage or subcutaneous collection at the 23rd postoperative day.

### 2.6 Follow‐up

Serial neurologic examination during 6‐months postoperative period was acceptable and no new neurological deficits were observed. We obtained serial OPD visit and brain CT scans with two episodes of brain and spine MRIs which revealed no hydrocephalus or hematoma and partial resolution of syringomyelia. The patient myelopathy and spastic gate improved significantly.

## 3 DISCUSSION

### 3.1 CSF circulation and pressure

Cerebrospinal fluid (CSF), as the protector of mammalian CNS, is mainly (75–90%) produced by choroid plexus of lateral ventricles and other structures such as brain parenchyma, ependymal cells, and interstitial fluid. CSF flows from lateral ventricles to third ventricle though foramina of Monro. Then, passing through cerebral aqueduct to the fourth ventricle and leaves it from (lateral) foramina of Lushka to cerebral cisterns and SAS; and (medial) foramen of Magendie to spinal SAS. The CSF is absorbed at Superior Sagittal Sinus (SSS) arachnoid granulations and Virchow–Robin space of peripheral SAS.^23^, ^24^ Any change in CSF production, absorption, or circulation can alter intracranial pressure (ICP) and therefore cerebral perfusion pressure (CPP) and cerebral blood flow (CBF) autoregulation.^23^, ^25^

### 3.2 Syringomyelia

Syringomyelia is classified into communicating and non‐communicating types.^26^ The CSF is normally flows into the SAS and its flow to the central canal is considered minimal.^23^, ^24^, ^25^ There is also a pressure variance between the fourth ventricle and the central spinal cord canal which results in CSF pulsatile flow from the ventricle to the central canal. The CSF flow in CVJ is bidirectional: from fourth ventricle to the central canal and vice versa.^23^, ^24^, ^25^, ^26^ Communicating syringomyelia is referred to backflow resistance of CSF from spinal central canal or SAS to cranial regions. Etiologies include trauma, infection, iatrogenic (postsurgical), and thecal sac injections or manipulations.^4^, ^5^, ^9^ Radiological characteristics include upward extension of syrinx to uppermost cervical segments, CSF flow in dynamic imaging, and contrast passage to syrinx cavity.^9^, ^26^

### 3.3 Adhesive arachnoiditis

Spinal adhesive arachnoiditis (SAA) is referred to formation of multiple arachnoid membranes in SAS due to various pathologies, mainly caused by inflammation.^1^, ^5^ It can lead to neural structures compression due to tethering, mass effect and alteration in CSF circulation and dynamics.^2^, ^3^, ^4^ In CVJ, mainly due to TB and sarcoidosis, it has been associated with syrinx formation and tethering of cerebellum and brainstem leading to neurological manifestation.^2^, ^3^, ^4^, ^5^

### 3.4 Our case and hypothesis

Herein we presented our case of foramen magnum SAA due to previous traumatic brain injury, forming numerous arachnoid membranes in CVJ, altering CSF circulation in CVJbeside local accumulation of CSF in dorsal aspect of FM with compression effect on brainstem and fourth ventricle. It obviously caused resistance in CSF flow and higher‐pressure gradient between cranial and spinal regions, documented in CSF dynamic study. Decompression of FM and release of arachnoid adhesions, are mainstay of SAA of the SVJ being associated with improved clinical outcome and resolution of the syrinx.^26^ The patient developed supratentorial SDH that was surgically evacuated. The proposed mechanism of this phenomenon might be rapid evacuation of the CSF from the axis, leading to subdural vein sagging and increased venous pressure followed by rupture of the veins and bleeding into the subdural space. The mechanism of the SDH formation in patients undergoing CSF diversion or drainage is similar to our case. Prevention of the CSF rapid drainage and replacement of the CSF with saline could be a prophylactic strategy in these patients.

We assumed that CSF dynamics in these patients adapt with a higher‐pressure gradient; explaining that CSF flow resistance in CVJ, causes higher cranial and lower spinal CSF pressure. CSF outflow and absorption adaptations, might cause higher cranial CSF pressure without ventriculomegally or other imaging findings of intracranial hypertension similar to normal pressure hydrocephalus (NPH) or idiopathic intracranial hypertension (IIH).^23^, ^24^ Because of this compensation, brain circulation and autoregulation adapts to higher CSF pressures as well. Thus, these conditions are associated with higher arterial blood pressure to maintain CPP and therefore higher venous pressure to sustain normal venous flow. It is recommended to prevent rapid CSF drainage from CVJ during surgery using cotton pads or filters to avoid catastrophic events such as supratentorial SDH.

## 4 CONCLUSION

Spinal adhesive arachnoiditis (SAA), caused by various etiologies leads to alterations in CSF circulation and neural structure tethering or compression. In CVJ, beside brainstem and cerebellum compression, spinal syrinx formation is a common sequela which is associated with scoliosis and neurologic deficits. Foramen magnum decompression and arachnolysis with direct observation of CSF flow from fourth ventricle to spinal SAS, is considered the standard of care. Posterior fossa decompression with abrupt CSF drainage is associated with cerebral cortical vein sagging and subsequent supratentorial SDH formation. We recommend in similar cases, to avoid rapid CSF drainage while performing durotomy by utilizing cotton pads and lowering the head level to avoid such catastrophic complications.

## AUTHOR CONTRIBUTIONS

**Keyvan Eghbal:** Conceptualization; methodology; project administration; writing – review and editing. **Majid Reza Farrokhi:** Conceptualization; resources; supervision; writing – review and editing. **Seyed Reza Mousavi:** Data curation; investigation; supervision; writing – review and editing. **Mohammadhadi Amir Shahpari Motlagh:** Investigation; software; validation; writing – review and editing. **Ali Kazeminezhad:** Data curation; formal analysis; software; visualization; writing – review and editing. **Fariborz Ghaffarpasand:** Data curation; formal analysis; investigation; validation; writing – original draft; writing – review and editing.

## CONFLICT OF INTEREST STATEMENT

None of the authors have any conflict of interest to declare regarding the manuscript.

## ETHICAL APPROVAL

All procedures performed were under the institutional and/or national research committee's ethical standards and the 1964 Helsinki Declaration and later amendments or comparable ethical standards. Shiraz University neurosurgery department board members supervised and approved this report on behalf of the Ethical Committee of Shiraz University of medical sciences (SUMS).

## CONSENT

The patient and their legal guardians provided their written consent for publication of the manuscript and the case for research purposes.

## Data Availability

Data and original images in the current study are available from the corresponding author on reasonable request. Authors can confirm that all relevant data are included in the article and/or its supplementary information files.
